# Supplementary material for: Comprehensive Genomic Characterization of Staphylococcus aureus Isolated from Atopic Dermatitis Patients in Japan: Correlations with Disease Severity, Eruption Type, and Anatomical Site
Source: Microbiol Spectr. 2023 Jul 11;11(4):e05239-22. doi: 10.1128/spectrum.05239-22 (PMC10434064; doi:10.1128/spectrum.05239-22)
Supplement: Supplemental file 2 — Supplemental material. Download spectrum.05239-22-s0002.pdf, PDF file, 0.8 MB [file spectrum.05239-22-s0002.pdf]

Supplemental Materials

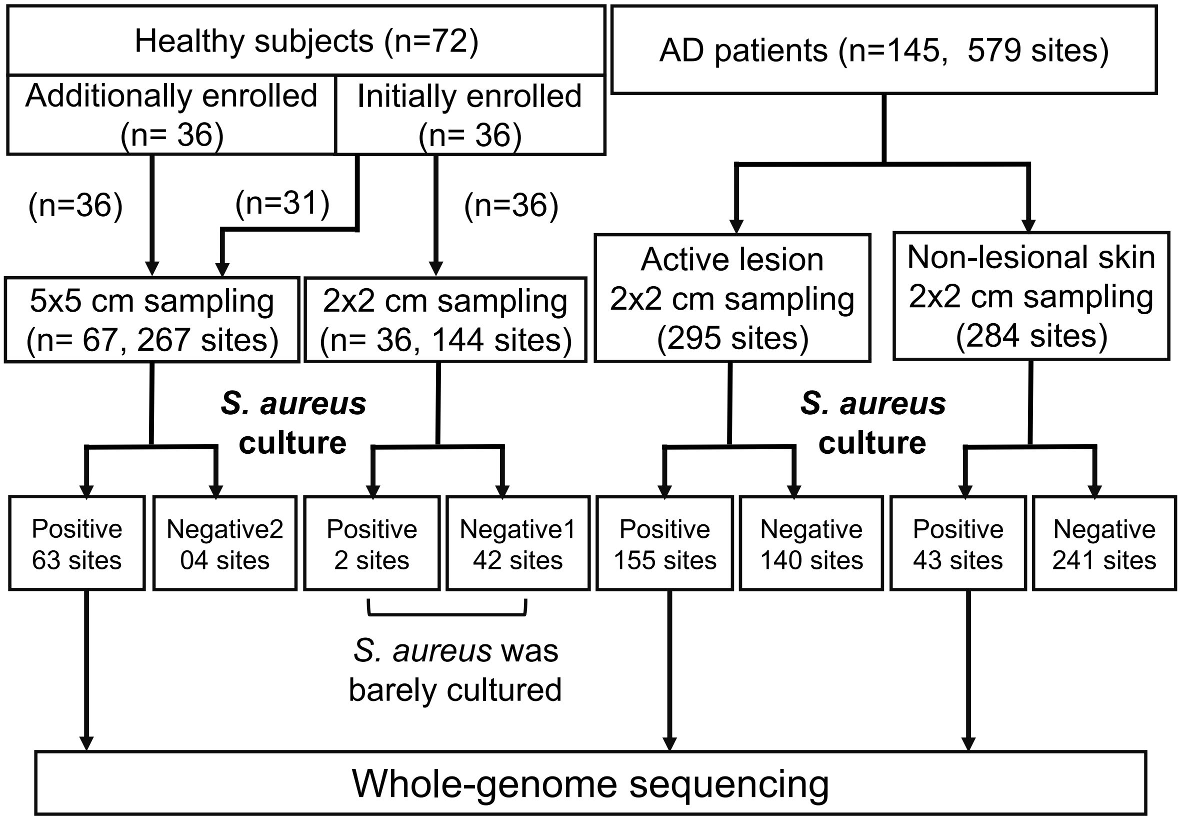

FIG S1 Schematic flow of the sampling, culture, and genotyping procedures. Swab samples (4 cm^2) were collected to evaluate the culture rate and genotypes of *Staphylococcus aureus* in atopic dermatitis skin with active lesions (AD-A), non-lesional atopic dermatitis skin (AD-NL), and healthy skin samples. However, *S. aureus* cultures from the 4 cm^2 healthy skin samples did not grow significantly. Therefore, the sampling area was expanded to 25 cm^2 for healthy skin to obtain an adequate sample quantity to determine *S. aureus* genotypes. In addition, 36 healthy individuals were enrolled in this study. *S. aureus* isolates cultured from 4 cm^2 areas of atopic dermatitis (AD) skin and 25 cm^2 areas of healthy individuals' skin were analyzed by whole genome sequencing (WGS).

Figure S2

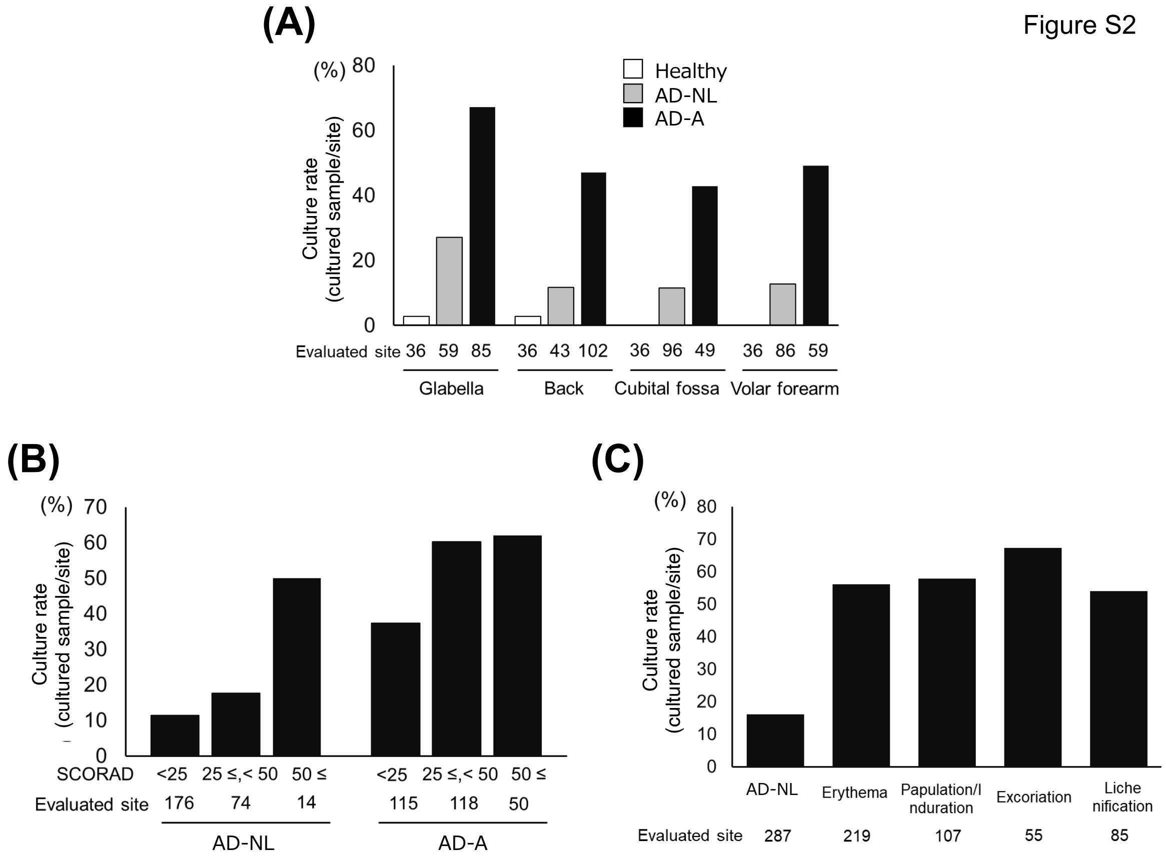

FIG S2 The culture rate of *Staphylococcus aureus*. (A) Culture rate of *S. aureus* in atopic dermatitis (AD) and healthy skin. *S. aureus* was cultured from samples collected from a 4 cm^2 area at 579 sites in 145 AD patients and 144 sites in 36 healthy individuals. (B) Culture rate of *S. aureus* in each lesion. (C) Culture rate of *S. aureus* according to the scoring atopic dermatitis (SCORAD) score. (D) Culture rate of *S. aureus* according to the eruption type.

Figure S3

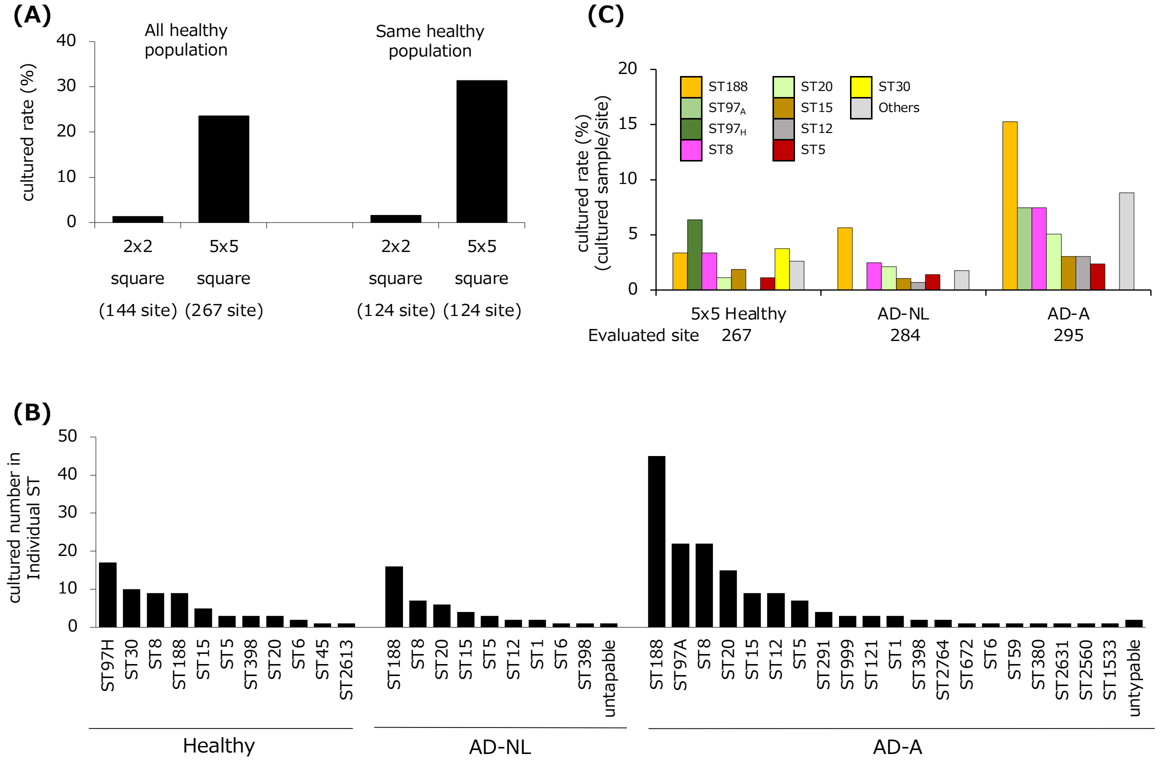

FIG S3 (A) Culture rates of *Staphylococcus aureus* in healthy skin. (B) The number of *S. aureus* sequence types (STs) cultured from healthy skin (25 cm^2; left), non-lesional atopic dermatitis skin (AD-NL; middle), and atopic dermatitis skin with active lesions (AD-A; right). (C) The culture rates of the identified *S. aureus* STs on healthy skin (25 cm^2; left), AD-NL (middle), and AD-A (right).

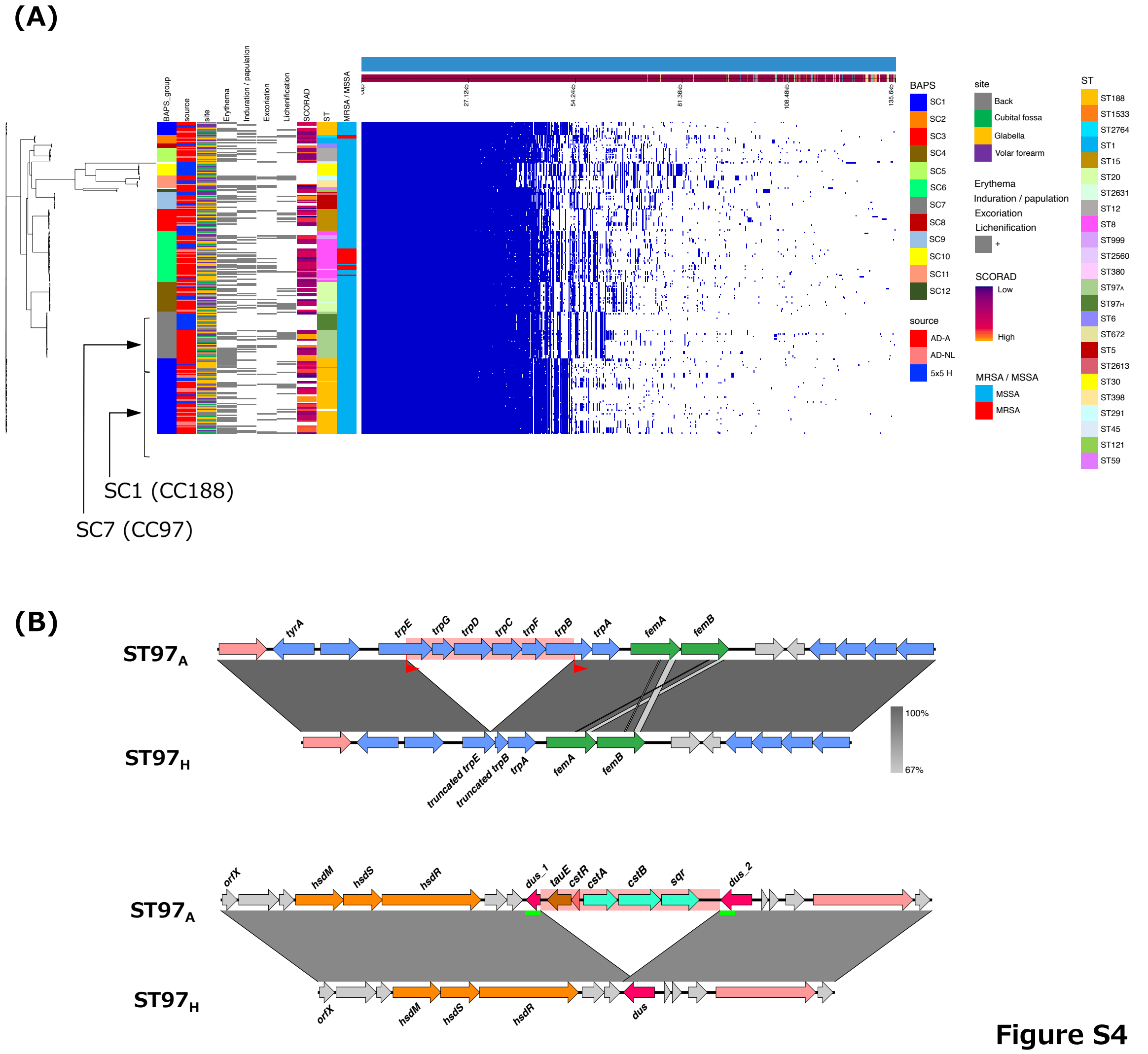

FIG S4 (A) Pan-genomic analysis of the 261 *Staphylococcus aureus* isolates and a phylogenetic tree of the 261 strains (left panel). The phylogenetic tree of genomes, as determined by Roary, compared to metadata and matrix, with the pan-genome presence (blue) and absence (white) of the core and accessory genes. (B) Comparison of the Trp biosynthesis operon and the sulfite-metabolism-associated region between the $ST97_A$ and $ST97_H$. Colored arrows of the Cluster of Orthologous Genes (COG) classification represent the coding sequences. Orange, defense mechanisms; red, RNA processing and modification; pale red, DNA replication, recombination, and repair; blue, amino acid transport and metabolism; green, cell wall/membrane/envelope biogenesis; and gray, function unknown.

In the upper panel, red arrowheads represent the direct repeats (DR1: AAAATATGAA and DR1: AAAACATGAA). In the lower panel, light green boxes represent the homologous regions. *cstA*, coding of persulfide response sulfurtransferase; *cstB*, coding of persulfide dioxygenase-sulfurtransferase; and *sqr*, type II sulfide: quinone oxidoreductase.

Figure S5

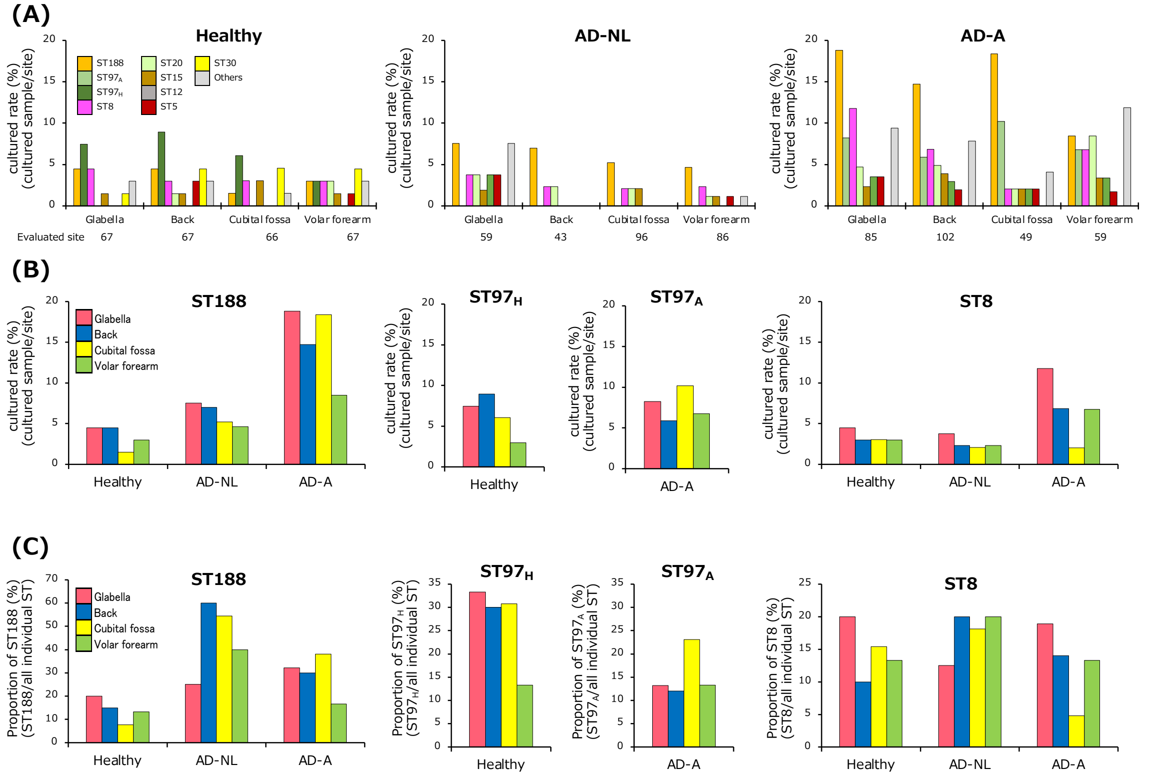

FIG S5 (A) Culture rates of *Staphylococcus aureus* sequence types (STs) from healthy skin (25 cm^2), non-lesional atopic dermatitis skin (AD-NL), and atopic dermatitis skin with active lesions (AD-A). The right panel represents healthy skin, the middle panel represents AD-NL, and the left represents AD-A. (B) The culture rates of ST188 (right panel), ST97 (middle panel), and ST8 (left panel) from healthy skin (25 cm^2), AD-NL, and AD-A. (C) The proportions of ST188 (right panel), ST97 (middle panel), and ST8 (left panel) from healthy skin (25 cm^2), AD-NL, and AD-A.

Figure S6

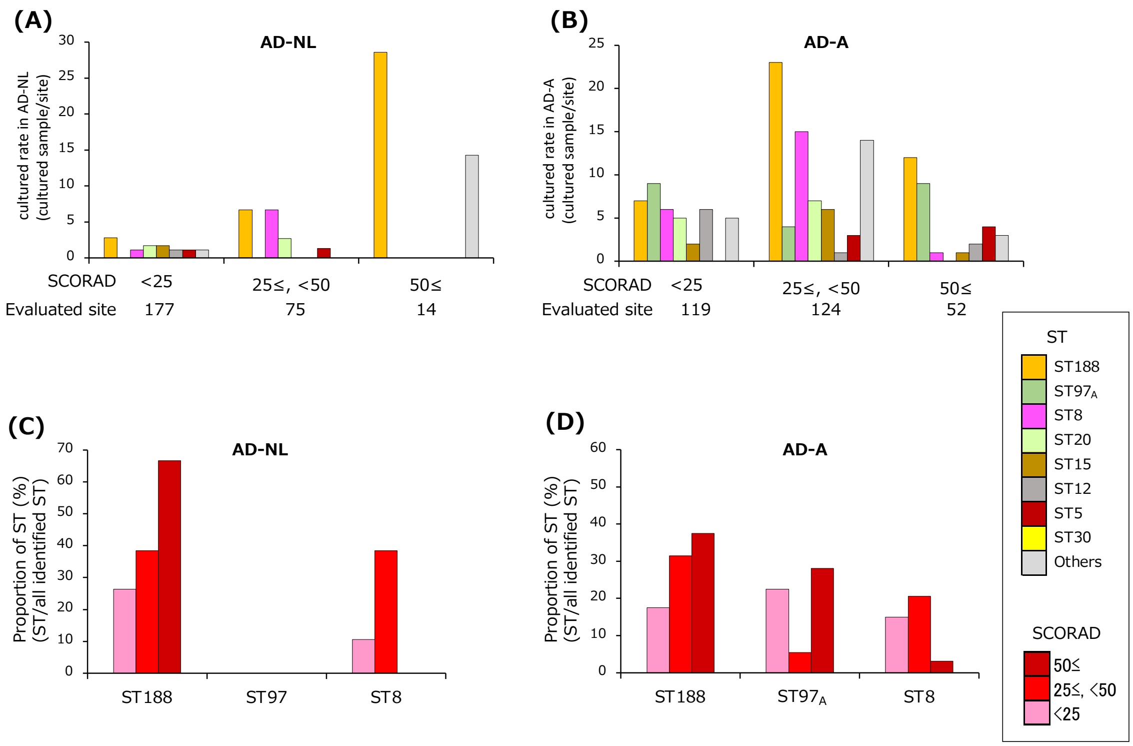

FIG S6 (A) Sequence type (ST) culture rates identified in non-lesional atopic dermatitis skin (AD-NL) according to disease severity, stratified according to the scoring atopic dermatitis (SCORAD) scores. (B) Culture rates of the identified STs in atopic dermatitis skin with active lesions (AD-A) according to disease severity, stratified according to SCORAD scores. (C) Culture rates of ST188, ST97, and ST8 in AD-NL according to the disease severity. (D) Culture rates of ST188, $ST97_A$, and ST8 in AD-A according to the disease severity.

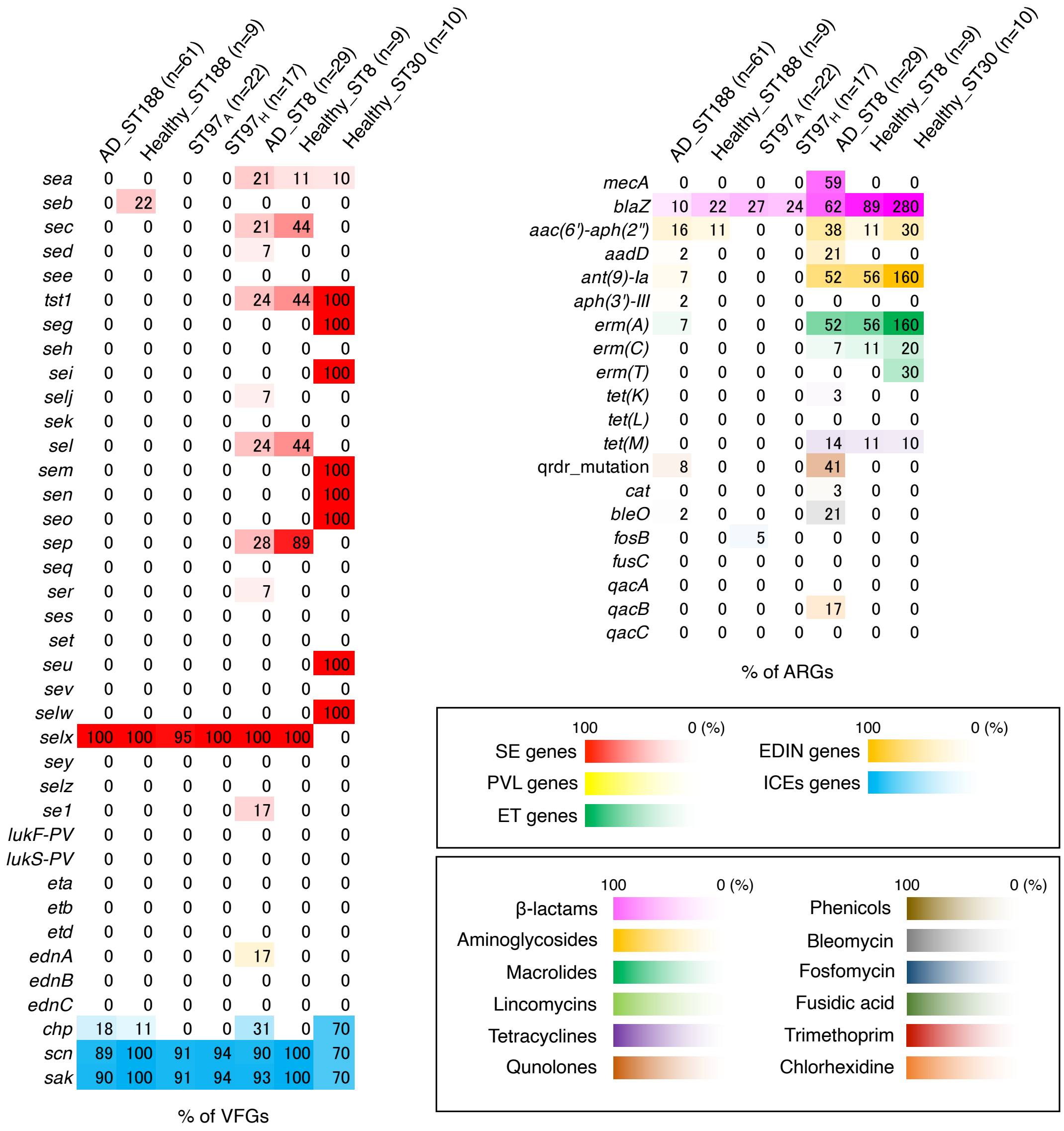

Figure S7

FIG S7 Characteristics of virulence factor gene (VFG) or antimicrobial resistance gene (ARG) patterns from atopic dermatitis (AD) skin- or healthy skin-derived sequence types (STs). (A) Heatmaps indicate the proportion of several VFGs and ARGs, and the colors represent each category.
